# Effect of Short Carbon Fiber Reinforcement on Mechanical Properties of 3D-Printed Acrylonitrile Butadiene Styrene

**DOI:** 10.3390/polym15092011

**Published:** 2023-04-24

**Authors:** Evgeniy Lobov, Anastasia Dobrydneva, Ilia Vindokurov, Mikhail Tashkinov

**Affiliations:** Faculty of Applied Mathematics and Mechanics, Perm National Research Polytechnic University, Perm 614990, Russia; eslobov@pstu.ru (E.L.); a.dobrydneva@pstu.ru (A.D.); ivv@pstu.ru (I.V.)

**Keywords:** 3D printing, ABS, short carbon fiber, mechanical tests, FE model, elastic characteristics, strength, fracture toughness

## Abstract

The effect of short carbon fiber (SCF) filler on the mechanical properties of 3D-printed acrylonitrile butadiene styrene (ABS) was investigated. The fused filament fabrication (FFF) method was used for the manufacturing of samples. Elastic properties and strength characteristics of samples made of conventional ABS and SCF-reinforced ABS were compared in tensile and bending tests. Fracture toughness and critical strain energy release rate were also determined. In addition, 3D-printed monofilament SCF-reinforced samples were fabricated, the internal structure of which was analyzed using microcomputed tomography (micro-CT). Based on the tomography data, finite-element (FE) models of representative volume elements (RVEs) of the reinforced material were created and used for the numerical calculation of effective characteristics. Numerical and experimental results for the effective elastic properties were compared with the Mori-Tanaka homogenization technique. The ABS samples filled with SCF showed considerably higher mechanical characteristics than those of the conventional ABS. Finally, the dependence between the strength characteristics and elastic properties of the samples on the diameter of the nozzle used for 3D printing was established. 3D-printed ABS reinforced with SCF demonstrated a gain in tensile strength and fracture toughness by 30% and 20%, respectively. Interlayer adhesion strength in flexure tests showed an increase of 28% compared to pure ABS samples.

## 1. Introduction

Additive manufacturing in the form of 3D printing based on layer-by-layer material deposition is currently used for manufacturing parts with complex geometry [1]. One of the most accessible methods of 3D printing is fused filament fabrication (FFF), which works with polymeric materials in the form of a filament [2,3]. A large variety of thermoplastic polymers were adapted to be used with this method. In addition to the initial material properties, the effective mechanical characteristics of the manufactured products are influenced by printing parameters, such as nozzle diameter, infill pattern and angle, infill density, printing speed and others [4], as well as different types of postprocessing treatment [5,6]. Additive composites with a polymer matrix and natural or synthetic microscale additives were developed to improve stiffness, elastic modulus and strength of 3D-printed polymers [7,8,9]. In particular, short carbon fibers [10,11,12,13,14,15], as well as continuous carbon fibers Field [16,17], are widely used as the most promising reinforcing agents.

Various issues related to thermoplastic polymers with randomly distributed SCF were addressed in many papers. For example, according to the latest studies, short carbon fibers were introduced into the designed 3D orthogonal preforms, which compressive behavior was characterized in [18]. The tensile strength, elastic modulus and fracture toughness of 3D-printed samples of SCF-reinforced ABS were studied in [19,20,21]. The interlayer mode-I fracture toughness of FFF-printed SCF-reinforced ABS materials was examined in [20] using a modified double cantilever beam (DCB) test. Paper [22] presents results of the interfacial bonding strength between printed wires of ABS, carbon nanotube-reinforced ABS and SCF-reinforced ABS specimens. The overheat FFF printing was used in [23] to fabricate ABS composite specimens with enhanced mechanical performance. The effect of carbon fiber concentration and type, infill pattern and environmental temperatures on the mechanical properties of the printed polyamide samples were investigated in [24]. The results of [25] indicated that the gradual change in fiber reinforcement reduced the stress concentrations at the interface zone and increased the strength of ABS. The numerical methods were also applied to predict mechanical properties and analyze the effect of various compositions and manufacturing parameters. Mechanical properties of SCF-reinforced PLA specimens were predicted using three-scale asymptotic homogenization for both random and aligned fiber distributions [26]. In [27], attention was given to the impact of process parameters from the micro- to macro-level by integrating image-based statistical analysis with physics-based modeling.

Although additive composites with SCF have been successfully fabricated and are commercially available, understanding their deformation and fracture mechanisms is a relevant subject of research because of the complexities of the microstructural morphology. In particular, the presence of microscale defects in the form of matrix voids, fiber damage and irregular fiber orientation can alter the targeted properties of these composites.

The aim of this work is to investigate the effect of manufacturing parameters and resulting microstructural characteristics on the elastic and fracture properties of 3D-printed SCF-reinforced ABS samples by comparing the results of experimental and numerical studies. The mechanical properties of samples were evaluated through a series of tensile and bending tests. The internal microstructure was examined using micro-CT scans and images from scanning electronic microscopy (SEM). Finite element simulations were performed to assess the effective response of RVEs based on the internal structure of the real monofilament samples. Some novel testing procedures and methods were suggested and implemented. New results on stiffness and strength properties of SCF-reinforced printed samples were obtained and compared with those made of standard ABS material.

## 2. Materials and Methods

### 2.1. Samples Manufacturing

**Figure 1:**
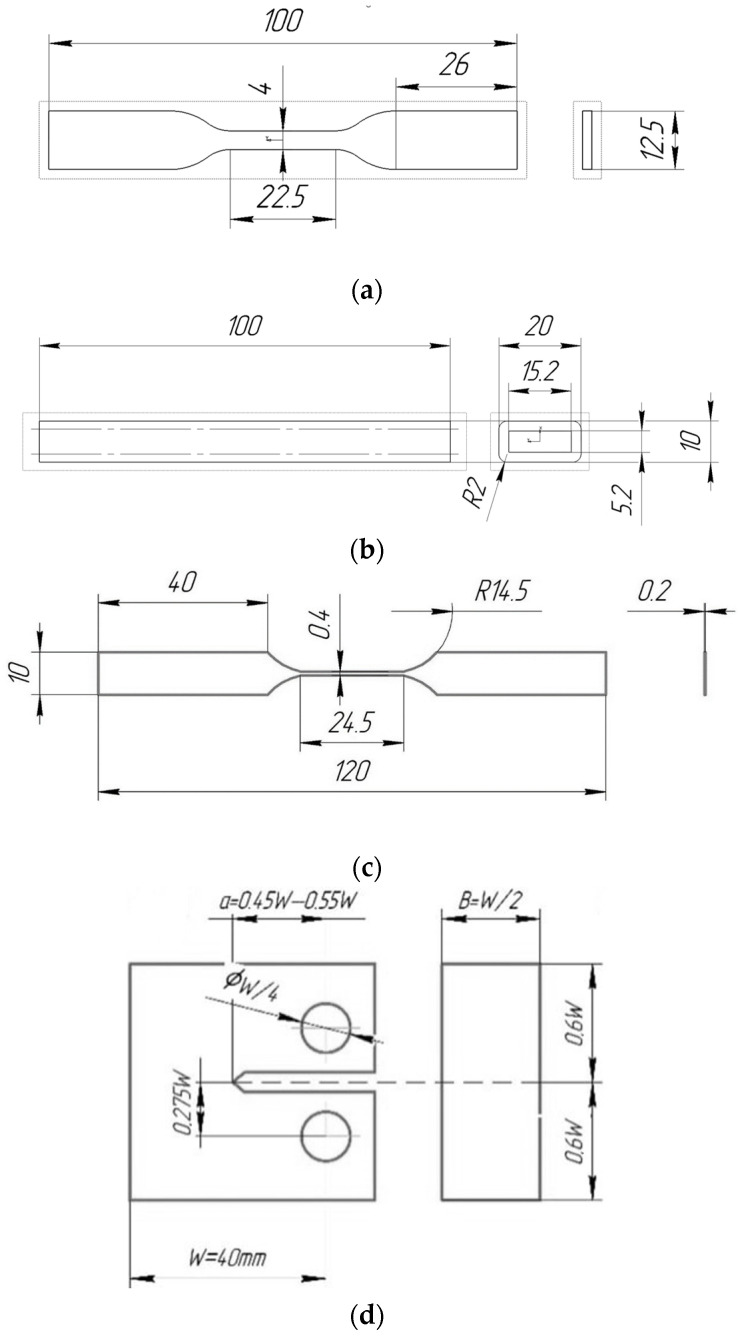
Dimensions of samples: (**a**) for tensile tests; (**b**) for bending tests; (**c**) monofilament tensile tests, (**d**) Geometry of CT sample.

A commercial ABS filament, as well as a filament with ABS matrix and SCF filler from REC (Russia) with a diameter of 1.75 mm, was used for the experiments. A Raise3D Pro2 Plus 3D printer was used for sample manufacturing. Standard samples for tensile and three-point bending tests, as well as monofilament samples for tensile tests, were produced with nozzle diameters of 0.4 mm and 0.8 mm. The tensile samples (Figure 1a) were made according to ISO 527-2:2012, with two infill angles: 0 and 90 degrees. The three-point flexural testing method was used to investigate the interlayer adhesion. The samples for bending tests were rods (length 100 mm, width 20 mm, height 10 mm) with an inner hole (length 100 mm, width 15.2 mm, height 5.2 mm). The size of the hole was chosen so that the wall thickness was divided by integer parts of the line widths when printing with different nozzles (Figure 1b). Single-layer monofilament samples were produced in such a way that the thickness of the working area of the sample corresponded to the thickness of the nozzle (Figure 1c).

**Figure 2:**
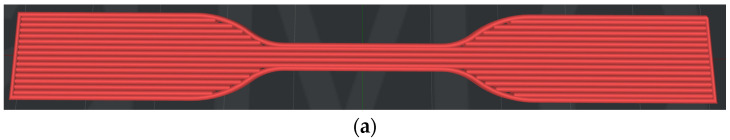

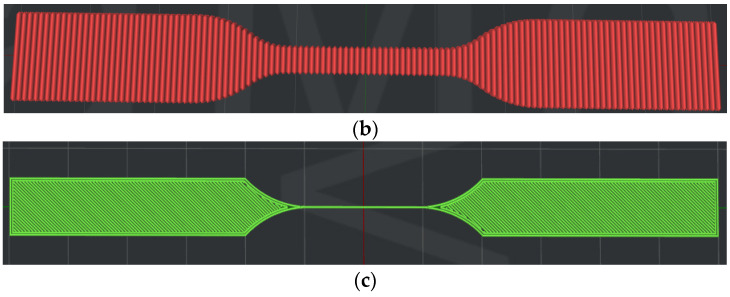
Tensile sample models and filling: (**a**) infill angle 0°; (**b**) infill angle 90°; (**c**) monofilament.

The samples were printed with the infill angle of 0° and 90°, as shown in Figure 2a,b. For SCF-reinforced samples, the following printing parameters were taken: layer thickness 0.2 mm, table temperature 110 °C, nozzle temperature 290 °C, infill/drawing ratio 100% straight, printing speed 30 mm/s. In the case of pure ABS, the table temperature (100 °C) and nozzle temperature (255 °C) were different. All samples were printed in a closed chamber and cooled to room temperature naturally.

### 2.2. Mechanical Characterization

Tensile tests were performed at a constant displacement rate of 0.5 mm/min and ambient temperature (~20 °C) on an Instron 68SC-5 universal testing machine with a 5 kN load cell. The accuracy of load measurement is 0.5% of the measured value in the range from 5 N to 5 kN, and the resolution of the movement is 0.0095 µm. An AVE2 video extensometer was used to measure displacements. Two points were marked on each sample surface at an equal distance from the sample middle plane, and the video extensometer was used to track the movement of these points to measure the elongation of the sample. The elastic tensile properties of the samples were obtained from the analysis of the elastic section of the stress-strain curve. In order to collect statistics, each of the sample configurations was tested at least 15 times to assess repeatability.

Bending tests were performed at a constant displacement rate of 0.1 mm/min with a 500 N load cell. The ratio used was:

(1) $$\sigma =\frac{6Flh}{4\left(bh^{3}-b_{1}h_{1}^{3}\right)}$$

where h is sample height, b is sample width, $h_{1}$ is the height of the interior part of a sample and $b_{1}$ is interior sample width.

**Figure 3:**
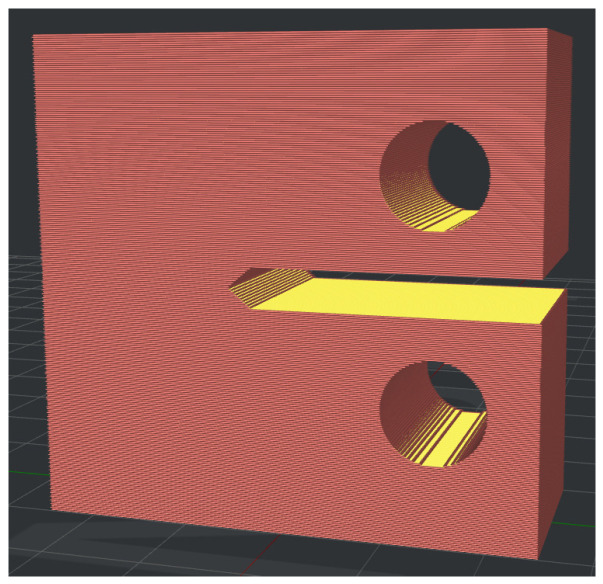
Compact tension (CT) sample model.

The tensile fracture properties were evaluated according to the ASTM D5045-14 standard for measuring the fracture toughness of polymers under plane strain state. Compact tension (CT) samples (Figure 3) were used. Their infill pattern and slicing parameters were chosen in a way that the crack propagated perpendicular to the printing layers stacking. The such configuration ensures the highest achievable fracture toughness value.

After manufacturing of CT samples, the supports in the crack area were removed using a table-top drilling and milling machine. A razor blade with a thickness of 0.08 mm was used to form a sharp notch marking the initial position of the crack. The crosshead speed of an Instron 68SC-5 universal testing machine during the tests was 1 mm/min. A VIC-3D Micro-DIC system (Correlated Solutions, Irmo, SC, USA) was used to measure the displacement field near the opening crack. Before the test, a white background and a black pattern were applied to the samples using an airbrush. Sequential speckle images were obtained using two 5.0-megapixel cameras to evaluate the displacement field. The average strain in the crack opening zone was estimated using Vic-3D 9 software (Correlated Solutions, Irmo, SC, USA). The fracture properties of the samples were obtained from the stress-strain curve.

### 2.3. Analysis of Microstructure

The surface of the samples was analyzed using an EM-30+ scanning electron microscope (COXEM, Daejeon, Korea) at magnifications of 100×, 200×, 500×, and 1000×. Before the morphological analysis, a thin layer of gold was disposed of on the samples. An accelerating voltage of 15 kV was used, and the analysis was performed using a secondary electron detector.

To study the internal microstructural features of additive composites, tomographic imaging of samples of the monofilament 3D printed samples was performed on a SkyScan 1272 Bruker micro-CT with the following parameters: X-ray tube voltage 37 kV, current 57 μA, resolution (voxel edge size) 1 μm, exposure time 3000 μs, sample rotation step 0.1° with 360° imaging with averaging over 4 frames. The duration of the sample scanning was 16 h and 45 min.

### 2.4. Numerical Methods

For numerical analysis of the elastic properties of an SCF-reinforced material, it is necessary to create a representative volume element (RVE)—a small material sample containing a sufficient statistical description number of microstructural elements of each component. These RVEs, together with information on geometry, volume fraction, fiber orientation, and fiber length distribution, were obtained from the micro-CT data. Each RVE was discretized using tetrahedral elements and subjected to uniaxial tensile loading. Calculations were performed in SIMULIA Abaqus 2022 (Dassault Systemes, Montréal, QC, Canada) with the C3D4 element type.

In addition to numerical calculations, analytical estimation of the effective elastic properties was performed with the Mori-Tanaka homogenization scheme [28], which gives estimates based only on the volume fraction and elastic properties of individual constituents.

## 3. Results

### 3.1. Strength and Stiffness

#### 3.1.1. Tensile Properties

**Figure 4:**
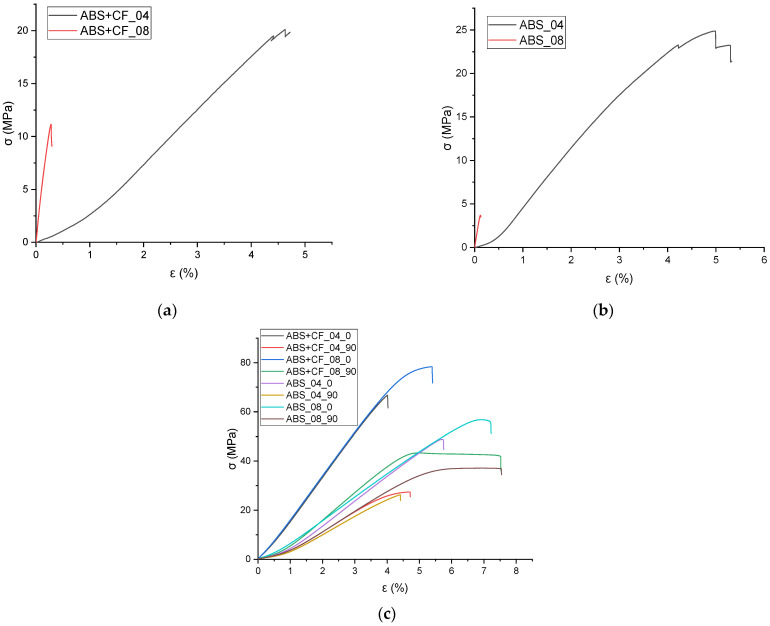
Tensile tests stress-strain diagrams for a series of samples: (**a**) ABS + CF monofilament printed with different nozzles; (**b**) ABS monofilament printed with different nozzles, (**c**) ABS + CF and ABS dog-bone samples printed with different nozzles and infill angles.

**Figure 5:**
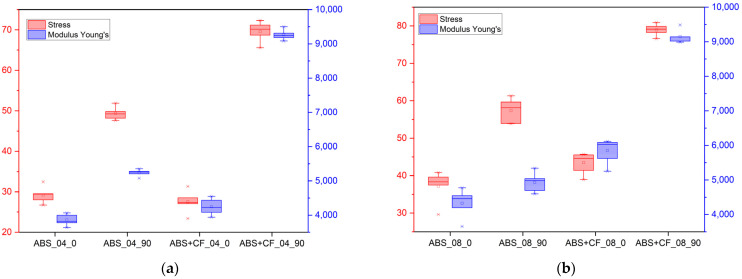
Tensile strength values and elastic moduli of samples printed with different nozzle diameters: (**a**) 0.4 mm; (**b**) 0.8 mm.

The tensile strength and Young’s modulus values grouped by material, nozzle diameter and sample type are shown in Table 1. The stress-strain curves obtained from the tensile tests are shown in Figure 4, and the corresponding block diagram is shown in Figure 5. The results are grouped according to the nozzle diameter and infill angle parameter. Hereinafter in tables and figures, ABS + CF, stands for SCF-reinforced ABS material. The accepted short notation also includes the used nozzle diameter and infill angle. E.g., “ABS + CF_04_90” means a sample of SCF-reinforced ABS, printed using a nozzle with a diameter of 0.4 mm and with a 90° infill angle.

The highest tensile strength (79.12 ± 1.6 MPa) was observed for ABS + CF samples printed with a 0.8 mm nozzle and 0° infill angle. The use of a 0.4 mm nozzle resulted in the highest tensile modulus value (9242.48 MPa) for the ABS + CF samples with a 0° infill angle (Table 1, Figure 5). The strength of pure ABS samples printed with a 0.4 mm nozzle and 0° infill angle was 67% higher than that of samples with a 90° infill angle. For pure ABS samples printed with a 0.8 mm nozzle, the difference between the strength of samples with different infill angles is 52%.

For ABS + CF, the strength upon change of an infill angle differs by 150% for samples manufactured with a 0.4 mm nozzle and by 77% for samples manufactured with a 0.8 mm nozzle. The maximum strength of the ABS + CF samples is 42.5% higher than that of pure ABS samples when printed with a 0.4 mm nozzle and 36% higher when printed with a 0.8 mm nozzle.

Similar trends were observed for the modulus of elasticity: samples with 0° infill angle exhibited stiffer behavior in all cases. However, in contrast to the strength characteristics, the samples printed using a nozzle with a smaller diameter have a higher (by 1–5%) stiffness at a 0° infill angle. For 90° infill samples, the elastic properties are significantly lower than those of 0° infill samples for both pure ABS and reinforced ABS.

Tensile tests of the monofilament samples showed ambiguous results—the strength characteristics of the samples printed with the 0.8 mm nozzle are significantly lower than those of the samples printed with the 0.4 mm nozzle (Figure 4). This is observed for both pure and reinforced materials. At the same time, on average, the elastic modulus of the monofilament exceeds that of the tested standard samples but has a large scatter of values.

**Figure 6:**
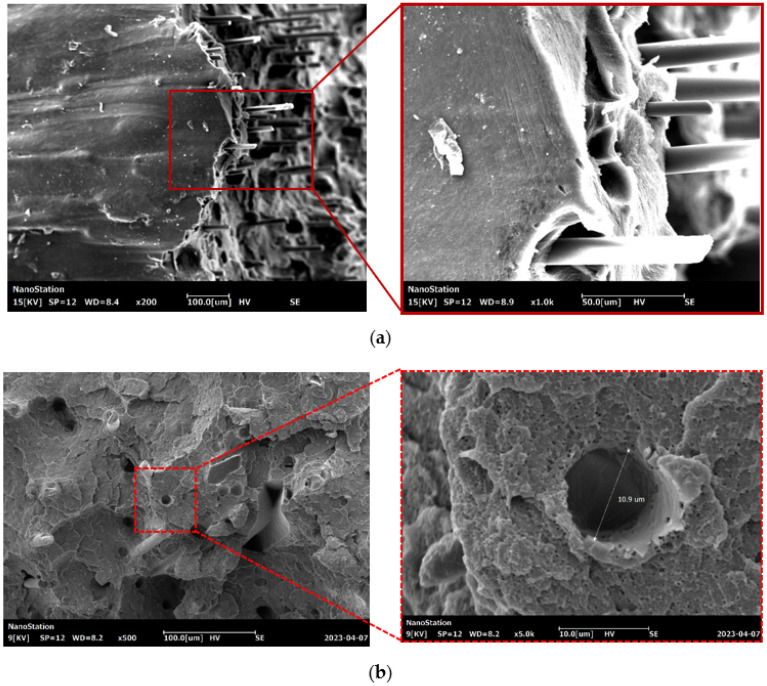
SEM images of the surface: (**a**) side view on the fracture plane; (**b**) front view on the fracture plane.

Figure 6 shows SEM images of a surface of a fractured 3D printed ABS + CF monofilament sample after a tensile test at a magnification of 100×, 200×, 500× and 1000× times. Gaps are observed between the matrix and short fibers at the location of the fracture, indicating possible imperfect adhesion of the short fiber in the filament. The presence of defects in the form of free cavities can be related to the pullout of short fibers. Pulled short fibers in the field of vision have a smooth surfaces without any residual polymer material on them.

#### 3.1.2. Flexural Properties

**Figure 7:**
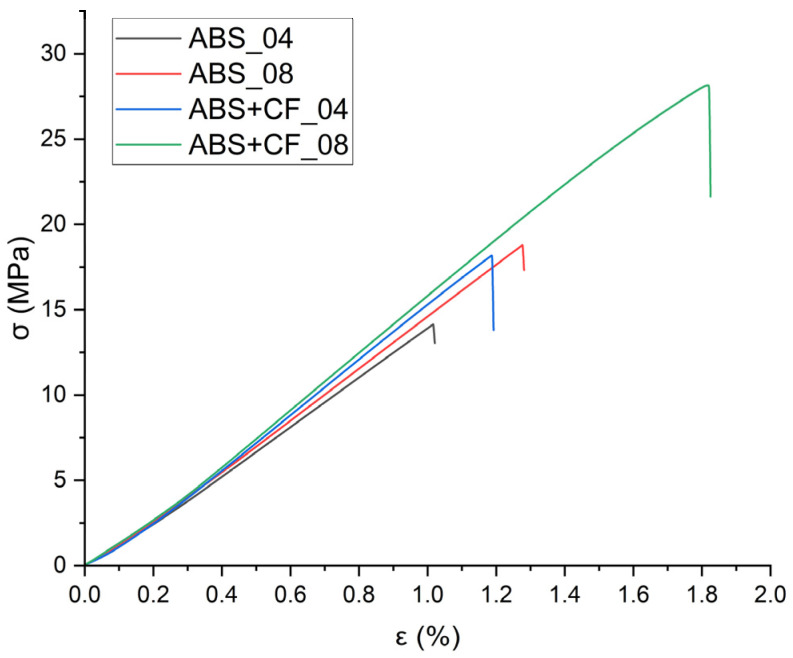
Bending tests stress-strain dependences for a series of samples printed with 0.4 mm and 0.8 mm nozzles.

**Figure 8:**
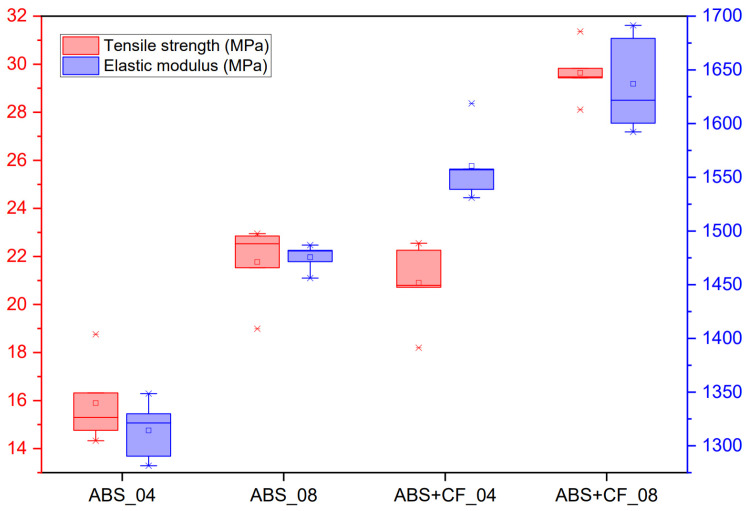
Bending strength and bending modulus of samples printed with different nozzle diameters.

Bending strength and bending modulus values grouped by material type and nozzle diameter are shown in Table 2. The stress-strain curves obtained from the flexural tests are shown in Figure 7, and the corresponding block diagram is presented in Figure 8. The highest bending strength was observed for samples printed using a 0.8 mm nozzle with a printing speed of 30 mm/s and a nozzle temperature of 290 °C. In the three-point bending test, all printed samples first showed linear elastic deformation and then reached the maximum bending stress. Samples printed with the 0.8 mm nozzle showed higher deformation than samples printed with the 0.4 mm nozzle while maintaining a linear behavior. SCF reinforcement does not solve the problem of the influence of nozzle diameter on the interlayer sintering and strength of the samples: in the case of pure ABS, the difference between the 0.4 mm and 0.8 mm nozzle samples was 32%, and 42% in the case of ABS + CF. The flexural modulus is 12% higher for ABS samples made with a 0.8 mm nozzle and 4% higher for ABS + CF samples.

#### 3.1.3. Fracture Toughness and Critical Strain Energy Release Rate

Fracture toughness (critical stress intensity factor) $K_{Ic}$ of the first fracture mode was determined by formula according to the method given in ASTM D5045 [29,30]:

(2) $$K_{Ic}=\frac{P_{Q}}{BW^{\frac{1}{2}}}f\left(x\right)$$

where $P_{Q}$ is load, *B* is sample thickness, *W* is sample width, *a* is crack length, x=a/W,

(3) $$f\left(x\right)=\frac{\left(2+x\right)\left(0.886+4.64x-13.32x^{2}+14.72x^{3}-5.6x^{4}\right)}{{\left(1-x\right)}^{\frac{3}{2}}}$$

The critical strain energy release rate then can be determined as [29,30]:

(4) $$G_{Ic}=\frac{\left(1-\nu^{2}\right){K_{Ic}}^{2}}{E}$$

where *E* is elastic modulus, obtained during fracture toughness tests, *ν* is Poisson’s ratio, $K_{Ic}$ is critical stress intensity factor. The adopted scheme assumes linear elastic fracture.

The criterion described in Section 9.1.1 of the ASTM D5045 [29] was used to ensure the reliability of $K_{Ic}.$ In addition, the sample size was chosen according to this criterion, which ensured a flat deformed state at the apex of the crack.

The ABS + CF sample made with a 0.4 mm nozzle showed better fracture toughness (by 28%) and critical strain energy release rate (by 48%) characteristics compared to conventional ABS (see Table 3). Crack propagation in the samples was sequential, with delamination along the layers. The crack tip in samples with compact tension has a higher probability of advancing across the layer-layer interface, where the interfacial zone between the layers acted as the weakest link, contributing to softening. This effect was stronger for pure ABS samples.

**Figure 9:**
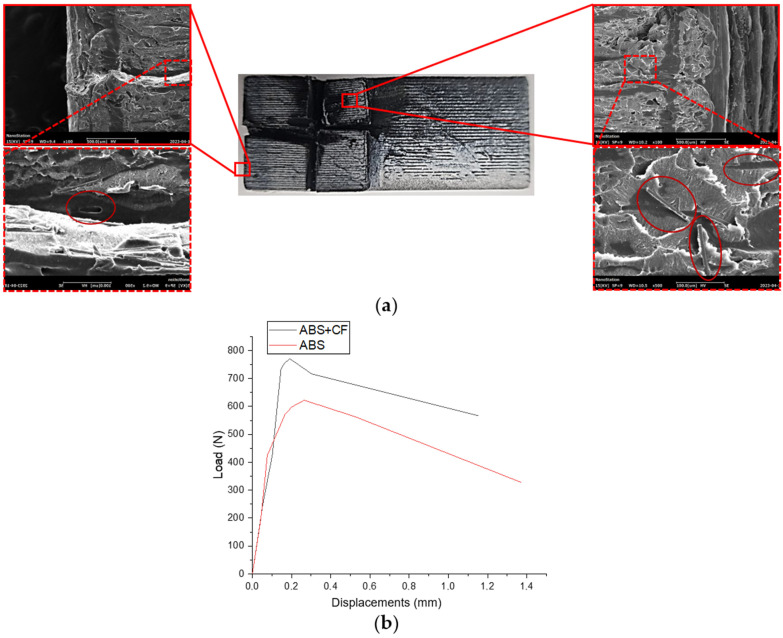
(**a**) SEM images of fractured CT-sample printed with ABS + CF, two sections at the start and at the end of crack formation are zoomed in; (**b**) force-displacement diagrams for CT samples tension tests averaged over a series of samples.

Figure 9a demonstrates SEM images of the fractured ABS + CF CT-sample surface. A number of randomly oriented short fibers imprinted in the matrix, as well as fibers pulled out from the matrix, are clearly visible on the surface. Although the crack plane is perpendicular to the printing layers stacking, there were no abrupt changes in the direction of crack propagation. Force-displacement diagrams in Figure 9b show differences in the mechanical behavior of 3D-printed CT samples made of pure ABS and SCF-reinforced ABS. The latter is able to withstand a tensile load of about 20% higher than the former.

### 3.2. Internal Structure Characterization

Computed tomography data was used for the generation of finite-element models of the material’s RVEs, which can be used for the investigation of mechanical properties and microscale mechanical behavior. This paper compares the effective elastic characteristics of these RVEs with the results of experimental studies of the samples, as well as with analytical estimates based on the mean-field method with a Mori-Tanaka homogenization technique.

**Figure 10:**
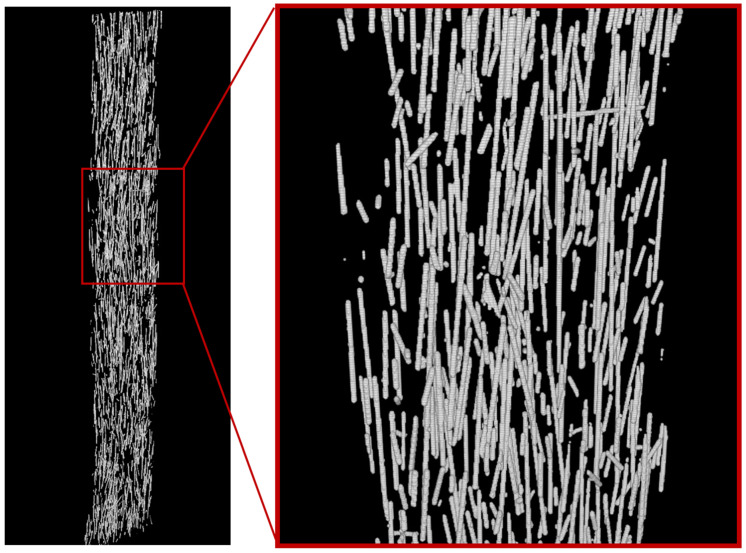
3D model of the fiber structure of a single-layer monofilament sample (general view and enlarged fragment) obtained from micro-CT data.

The obtained stacks of micro-CT X-ray images were used for stereological reconstructions and visualization of 3D models of the two-phase structure of 3D printed ABS + CF monofilament samples. Figure 10 shows a reconstructed model of a monofilament sample printed with a 0.4 mm nozzle. The average volumetric content of the short fiber in the sample, estimated from the 3D model, is 2.92%. The average diameter of the carbon fibers is 12 μm. The quantitative analysis of individual fibers was implemented to evaluate their parameters. The predominant number of fibers in the sample is less than 100 µm in length, which agrees with the results from [18], while there are single fibers longer than 400 µm also present. The fibers are predominantly oriented along with the printing direction, with single fibers located transversely. Deviation from the axis aligned with the printing direction in most cases does not exceed 15–20 degrees. Spatial distribution of fibers in the volume of the sample after the 3D-printing process remains uniform.

**Figure 11:**
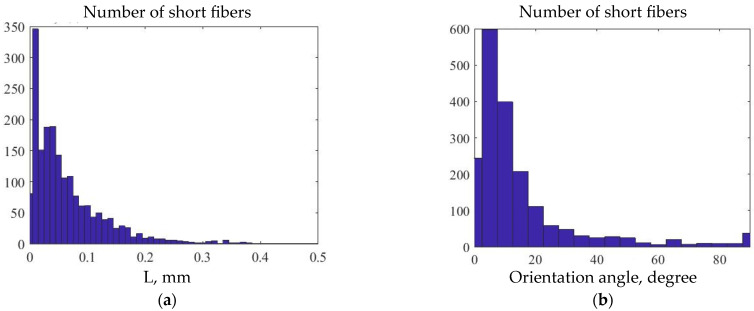
Histogram of fibers distribution in 3D-printed SCF-reinforced monofilament sample: (**a**) by fiber length; (**b**) by the angle of deviation from the vertical axis.

Figure 11 shows histograms of fiber distribution by length, as well as by the angle of their deviation from the axis coinciding with the printing direction in 3D-printed monofilament SCF-reinforced samples. Most of the fibers have lengths in a range between 0.01 mm and 0.2 mm, while their orientation angle is predominantly between 0 and 20 degrees.

### 3.3. Numerical Results

**Figure 12:**
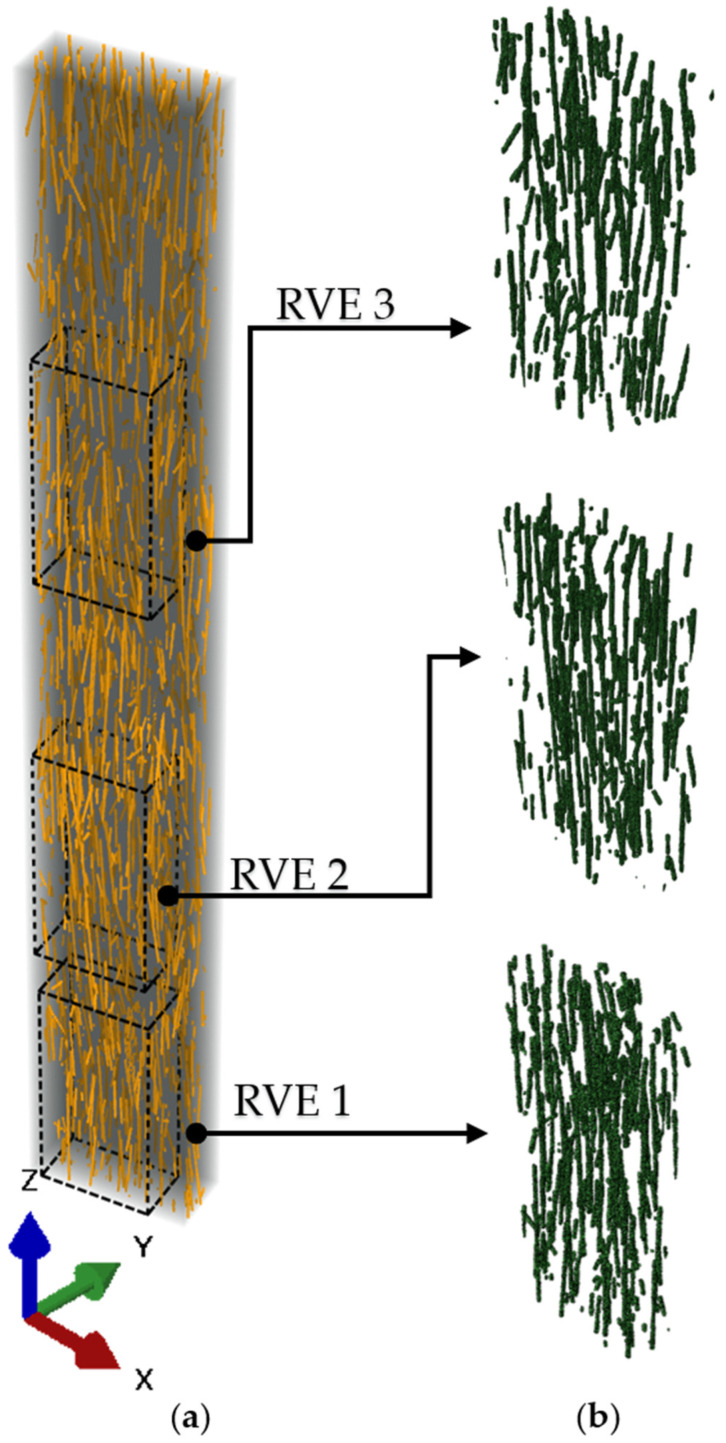
Three-dimensional model of the internal structure of monofilament sample printed with a 0.4 mm nozzle (**a**) and three RVEs selected from it (**b**).

Based on the micro-CT images, RVEs with dimensions X = 300 μm, Y = 160 μm, and Z = 600 μm were created. The dimensions were chosen in relation to the average fiber length: three fibers with maximal length fit in the Z dimension, which coincides with the printing direction, and, accordingly, is axial to the primary orientation of short fibers. According to the fiber length distribution analysis (Figure 6a), any RVE with the selected size will accommodate even the longest fiber from the distribution. The X and Y RVE dimensions were equal to 80% of the full monofilament sample size. Three areas were selected from the three-dimensional micro-CT model as RVEs, as shown in Figure 12.

The initial elastic characteristics of the matrix and SCF are presented in Table 4. The properties of the ABS matrix were obtained experimentally for samples printed with 0.4 mm and 0.8 mm nozzle and, in both cases, with 0° infill angle. The elastic properties and density of the carbon fiber were determined from other authors’ works [31,32].

To obtain the effective characteristics, RVEs were subjected to tensile loading with lower face fixed. Displacements of the upper face were restricted in the XY plane, and the load directed along Z axis was applied to the upper face. The effective Young’s modulus was determined by Hooke’s law:

(5) $$E=\frac{Fl}{S\Delta l}=\frac{F}{\varepsilon \times S},$$

where *ε* is the percentage of elongation, *F* is force, *S* is surface area.

Table 5 presents the results of the FE analysis and their comparison with the experimental data and with the results of the Mori-Tanaka homogenization.

The volume fraction of SCF in three investigated representative volumes of 3D-printed monofilament samples varied within the range from 2.81% to 2.96%. The Young’s moduli values obtained on the basis of FE-models for different RVEs from the same sample have shown insignificant deviation (up to 1.26%). The difference between Young’s moduli of the monofilament samples printed with different nozzle diameters is about 9%. Numerically calculated values of Young’s modulus differ from the experimental tension test values for monofilament printed samples on average by 46% in the case of a 0.4 mm nozzle and practically coincide in the case of a 0.8 mm nozzle. When comparing the numerically calculated values of the modulus with the properties of the standard tensile samples with 0° infill angle (see Table 1), 0.4 mm nozzle gives a 15.23% difference, while for 0.8 mm nozzle, it decreases to 4.43%. The Young’s modulus values obtained with the Mori-Tanaka homogenization technique differ downwards from the experimental values by 12% and from the results of the FE analysis by 3.5%. With different matrix properties defined by the samples printed with different nozzle diameters, the Mori-Tanaka model gives estimations with an 8% difference.

## 4. Discussion

An experimental study of the elastic moduli, tensile strength, and fracture toughness of samples of 3D printed SCF-reinforced was performed and compared to pure ABS. The dependence of the results on the nozzle diameter and infill angle was established. The obtained characteristics were compared to analytical predictions and the numerical results of the FE modeling of RVEs created using micro-CT three-dimensional data.

According to the tensile and flexural tests, the nozzle diameter was the most important parameter affecting the final mechanical properties of the printed samples. SCF reinforcement significantly increased the uniaxial tensile strength in samples with 0° infill angle (lines printed along load application). In the cases of transversal printing (90° infill angle, interlayer interaction between extrusion lines), SCF reinforcement does not give a clear increase in the tensile strength compared to pure ABS.

The numerical results from RVE models with morphology based on the micro-CT data predicts the effective elastic modulus better than the Mori-Tanaka homogenization technique, which, by definition, depends only on volume fraction, form, and properties of inclusions. This confirms that the parameters of short fibers, such as their length and alignment, have an effect on the elastic mechanical response of the additively manufactured composite material. However, in this particular case, results for Young’s modulus based on FE simulation and Mori-Tanaka estimations were quite close. On the contrary, experimental results for the monofilament samples were much higher than theoretically predicted. This means that not only microstructural parameters and distribution of SCF play a significant role, but also manufacturing process parameters and quality of the printed samples.

The diameter of the nozzle affects the printing process of the samples and, consequently, their effective mechanical properties. The width of the extruded filaments is indirectly determined by a nozzle diameter, but slicers allow some adjustments to these configurations [33]. Depending on the nozzle diameter, the number of printed lines required to fill the same area changes. Hence, the number of contact interfaces between printing lines changes as well. The surface area of these interfaces, as well as adhesion quality, play an important role and affect the resulting mechanical properties. Besides, in the case of short fiber reinforced polymers, some minor effects could be added by changing fiber orientation during the transition of the thermoplastic polymer through the nozzle. The less the nozzle diameter, the more pronounced this dependence.

The layer height of all samples was set to 50% of the nozzle diameter. The width of the contour lines and infill lines in the cross-sectional plane was set to 100% of the nozzle diameter. The tensile strength of samples printed with the 0.8 mm nozzle was generally higher than those of the 0.4 mm nozzle samples. This may be due to the larger sintering area of the layers printed with the 0.8 mm nozzle along the Y axis, which lead to an even distribution of stress along each line of the structure and prevents stress concentration. However, Young’s moduli were higher for samples printed with a 0.4 mm nozzle. This can be explained by the fact that samples printed with the smaller nozzle accommodate a greater number of material lines in the same volume. Oriented in the direction of the load, they redistribute this load better but have a lesser area of contact between printing lines which results in lower strength. In addition, other factors, such as temperature fluctuations of the layers due to the heater, fan, etc., during the experiment were not considered in this study but could also potentially have an effect on the resulting mechanical response.

## 5. Conclusions

The mechanical properties of 3D-printed samples made of pure ABS and SCF-reinforced ABS were compared. Tensile, flexural, and compact tension tests were performed. The standard, monofilament, and compact tension samples that were investigated in these tests were manufactured using nozzles of different diameters, as well as with two different infill angle patterns. The experimental results on the effective elastic properties were compared to the results of finite-element simulations and Mori-Tanaka analytical estimations, which showed sufficient agreement. FE models were created using the data from micro-CT characterization of the monofilament samples.

According to the results, adding short carbon fibers to the ABS matrix increased the tensile strength of the printed samples by 29.8%, that, combined with high-performance characteristics, such as surface quality and post-processability, opens up a wide range of possibilities for the solution of various engineering tasks using such material. Fracture and bending tests showed that the SCF-reinforced ABS performed well. However, the interlayer adhesion, as with the standard material, could be further improved. By selecting numerical simulation parameters, it is possible to create models that exploit the positive properties of short fiber reinforced polymer to the greatest extent possible.

## Figures and Tables

**Table 1:** Results of tensile tests.

	Nozzle Diameter, mm	Infill Angle	Tensile Strength (MPa)	Tensile Elastic Modulus (MPa)
ABS	0.4	$0^{{}^{\circ}}$	49.22 ± 1.66	5260.62 ± 101.45
		$90^{{}^{\circ}}$	29.43 ± 2.14	3819.44 ± 171.18
		Monofilament	26.94 ± 8.58	7648.32 ± 1077.95
	0.8	$0^{{}^{\circ}}$	58.14 ± 3.38	4978.21 ± 296.14
		$90^{{}^{\circ}}$	38.32 ± 4.41	4459.64 ± 427.69
		Monofilament	9.59 ± 7.41	6042.31 ± 119.20
ABS + CF	0.4	$0^{{}^{\circ}}$	70.14 ± 2.61	9242.44 ± 155.99
		$90^{{}^{\circ}}$	27.36 ± 2.87	4229.15 ± 248.56
		Monofilament	28.35 ± 4.32	11,709.16 ± 889.50
	0.8	$0^{{}^{\circ}}$	79.12 ± 1.65	9137.99 ± 197.26
		$90^{{}^{\circ}}$	44.62 ± 3.12	6023.59 ± 403.70
		Monofilament	12.51 ± 7.25	8704.72 ± 510.52

**Table 2:** Results of bending tests.

	Nozzle Diameter, mm	Bending Strength (MPa)	Bending Modulus (MPa)
ABS	0.4	15.30 ± 1.77	1321.25 ± 27.95
	0.8	20.23 ± 1.47	1481.46 ± 12.24
ABS + CF	0.4	20.80 ± 1.72	1556.99 ± 34.49
	0.8	29.47 ± 1.16	1621.77 ± 45.67

**Table 3:** Fracture toughness and critical strain energy release rate.

	$K_{Ic}$ MPa∗m^1/2^	$G_{Ic}$ kJ/m^2^	Strength (MPa)
ABS	1.28	0.031	0.62
ABS + CF	1.65	0.046	0.77

**Table 4:** Elastic mechanical properties of components.

Component	Density kg/m^3^	Young’s Modulus, MPa	Poisson’s Ratio
ABS matrix—sample printed with a 0.4 mm nozzle	1.05	5235.75	0.392
ABS matrix—sample printed with a 0.8 mm nozzle	1.05	5841.26	0.392
Carbon fiber	1.72	220,000	0.15

**Table 5:** Effective elastic characteristics of SCF-reinforced ABS monofilament sample obtained with different methods.

			Nozzle Diameter 0.4 mm	Nozzle Diameter 0.8 mm
FE analysis	Young’s modulus, MPa	RVE 1	8290	9035
		RVE 2	7955	8675
		RVE 3	7816	8540
Tensile test			11,709 ± 890	8704 ± 511
Mori-Tanaka estimation			7730	8414

## Data Availability

Data available on request due to restrictions eg privacy or ethical.
